# No advantage with navigated versus conventional mechanically aligned total knee arthroplasty—10 year results of a randomised controlled trial

**DOI:** 10.1007/s00167-022-07158-1

**Published:** 2022-09-27

**Authors:** Omer M. Farhan-Alanie, Tareq Altell, Sinead O’Donnell, Pauline May, James Doonan, Philip Rowe, Bryn Jones, Mark J. G. Blyth

**Affiliations:** 1 Department of Trauma & Orthopaedic Surgery, Glasgow Royal Infirmary, 84 Castle Street, Glasgow, G4 0SF UK; 2 Bioengineering Unit, University of Strathclyde, Graham Hills Building, 50 George Street, Glasgow, G1 1QE UK

**Keywords:** Knee arthroplasty, Randomised controlled trial, Computer Assisted Surgery, EM Navigation

## Abstract

**Purpose:**

Computer-assisted surgery (CAS) total knee arthroplasty (TKA) remains a controversial area of surgical practice. The aim of this study is to report the ten-year revision rates and patient-reported outcome measures (PROMS) of a single-blinded, prospective, randomised controlled trial comparing electromagnetically (EM) navigated and conventional TKA.

**Methods:**

199 patients were randomised to receive either EM navigated or conventional TKA where the aim of implantation was neutral mechanical alignment in all cases. Ten-year revision rates were collated and compared between the two intervention groups. Longitudinal PROMS data was collected prospectively at various time points up to 10 years post-operatively.

**Results:**

Over the ten-year period, there were 23 deaths (22.8%) in the EM navigation cohort and 30 deaths (30.6%) in the conventional cohort. At 10 years post-operatively, there was no statistically significant difference in all cause revision between the EM navigation and conventional cohort (4.0 vs 6.1%, *p* = 0.429). When analysing causes of revision that might be influenced by utilising EM navigation, there was no statistically significant difference in revisions (3.0% EM navigated vs 4.1% conventional group, *p* = 0.591). Patients that received navigated TKAs had improved Oxford Knee Society, American Knee Society Score and range of motion at 3 months following surgery compared to conventional TKA (*p* = 0.002, *p* = 0.032, and *p* = 0.05, respectively). However, from 1 to 10 years post-operatively, both interventions had equivalent outcomes.

**Conclusion:**

There is no difference in revision rates or clinical outcomes comparing EM navigated versus conventional TKA at ten-year follow-up. The expected mortality rate makes it unlikely that a difference in revision rates will reach statistical significance in the future. In the setting of an experienced knee arthroplasty surgeon, it is difficult to justify the additional costs of CAS in TKA surgery.

**Level of evidence:**

I

## Introduction

Computer-assisted surgery (CAS) total knee arthroplasty (TKA) remains a controversial area of surgical practice. Several studies have demonstrated improved precision of alignment with the use of CAS, allowing the surgeon to reproducibly implant a knee replacement to their alignment of choice [9, 14]. This has traditionally been targeted to within 3° of the mechanical axis, and this is based on studies of conventional knee replacement surgery that have demonstrated a higher rate of aseptic loosening if the components are implanted out with this range [12, 15, 16]. Whilst the logical assumption is that the use of CAS in TKA will improve implant survivorship, most studies have failed to demonstrate such benefit [13, 21]. Furthermore, there is currently no evidence to suggest that CAS TKA provides any improvement in patient-reported outcome measures (PROMs) [18, 28]. This calls into question not only the benefits of CAS but also the benefits of accurate neutral mechanical alignment. As a result there has been increasing focus on kinematic alignment in knee replacement surgery which aims to resurface the knee to its pre-disease state, with several studies demonstrating equivalent or improved patient-reported outcomes but with limited long-term survivorship data at present to support this  [3, 6, 29].

The iNAV Electromagnetic (EM) navigation system used in this study was developed to avoid the line of site problems encountered with infra-red systems, and the recurring contamination of the reflector balls on the reference arrays from blood and saw aerosols. The system employs small reference frames attached to the femur and tibia which are incorporated within the primary surgical incision, thereby avoiding the need for additional pin sites in the tibia and femur as required for infra-red trackers. This removes the potential complications of pin site infection and periprosthetic fracture related to the use of such bone pins [10].

The primary outcome of this randomised controlled trial (RCT) was to assess the precision of implantation of components comparing the iNAV electromagnetic system in TKA versus conventional TKA utilising standard instrumentation where the aim of implantation was for neutral mechanical alignment in all cases. The secondary outcome measures included PROMs, complications and all-cause revision. We have previously demonstrated that at one-year follow-up there was no statistically significant difference in the proportion of TKAs in either group implanted within 3° of the neutral mechanical axis, and no difference in component rotation in sagittal, coronal or axial planes as measured by computed tomography (CT) scan [2]. Mean tourniquet times were longer in the navigation group (80 vs 65 min, *p* = 0.001). There was no difference in the PROMs at either 1 or 5 years, but there appeared to be a trend in higher revision rate at five years in the conventional group compared to the navigation group (4.9 vs 0%, *p* = 0.08) [4]. The aim of this paper is to report the revision rates and PROMs at 10 years, with our hypothesis being that there would be no difference detected between the two interventions.

## Methods

### Participants

Patients were identified by members of the research team from TKA surgical waiting lists. Patients were invited to participate if they had osteoarthritis of the knee suitable for TKA, were able to provide informed consent and were aged 18 or over. There were no specific limits imposed on the degree of preoperative coronal or sagittal deformity.

### Randomisation

**Fig. 1:**
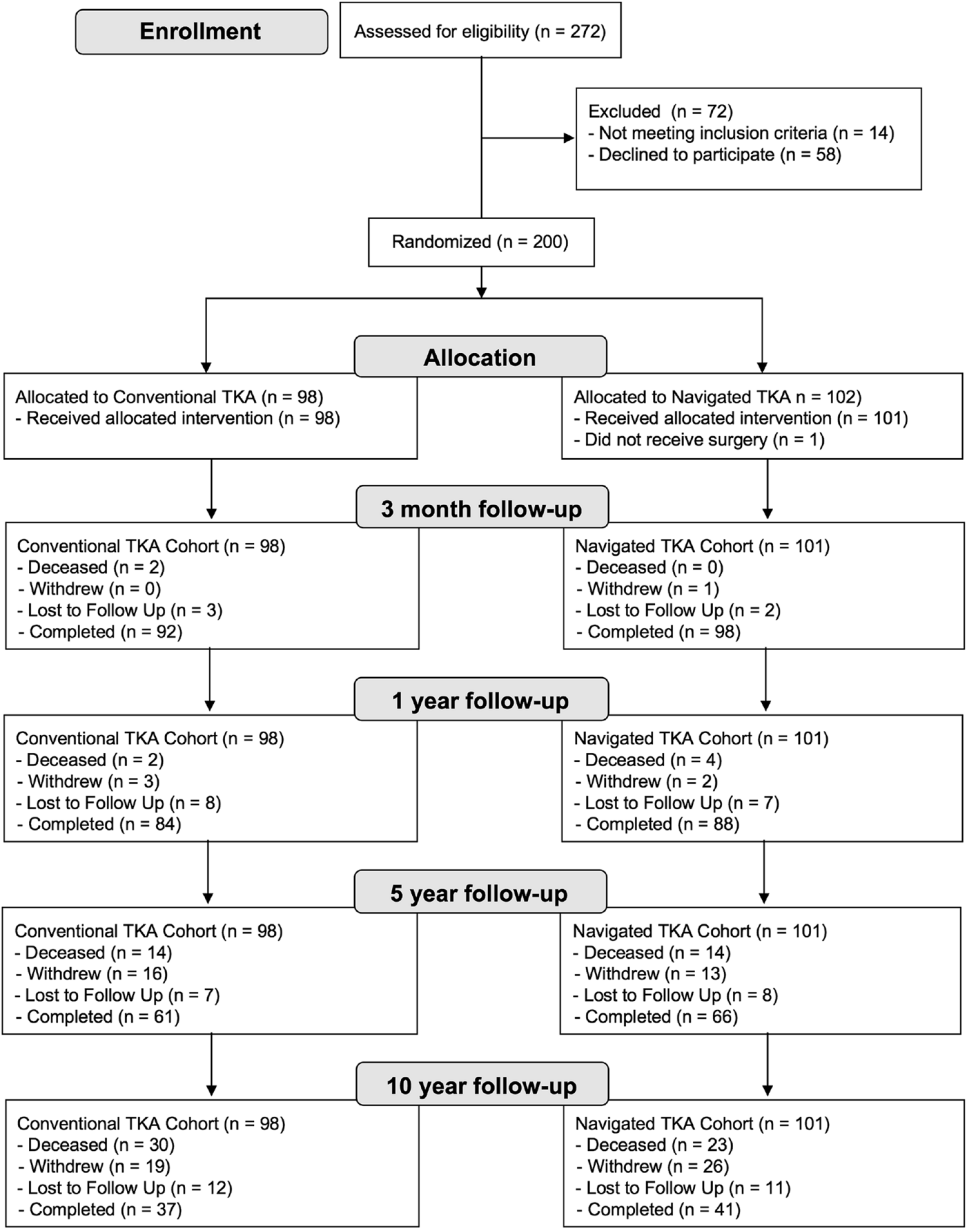
CONSORT (Consolidated Standards of Reporting Trials) Flow Diagram demonstrating the flow of patients through the randomised clinical study

Overall, 272 patients were screened between July 2007 and August 2010. Of the 272 screened patients, 14 were excluded for other medical reasons, and 58 participants decided that they did not want to participate in a research study. The remaining 200 patients were recruited and consented to the study giving a recruitment rate of 74% (Fig. 1). Patients were randomized in a 1:1 ratio to either navigated TKA or conventional TKA using a web-based computer-generated random number table. Randomisation was stratified by a surgeon to prevent surgeon bias and ensure that similar numbers of patients in each group were allocated to each surgeon. Randomization was successful in assigning equal preoperative patient demographics between the groups [2].

### Ethical approval

The study was approved by the Hospital Local Ethics Committee and the University Ethics Committee (07/S0704/6) and approved by our National Health Service (NHS) Trust Research and Development department before the commencement of the study.

### Surgical procedure

**Fig. 2:**
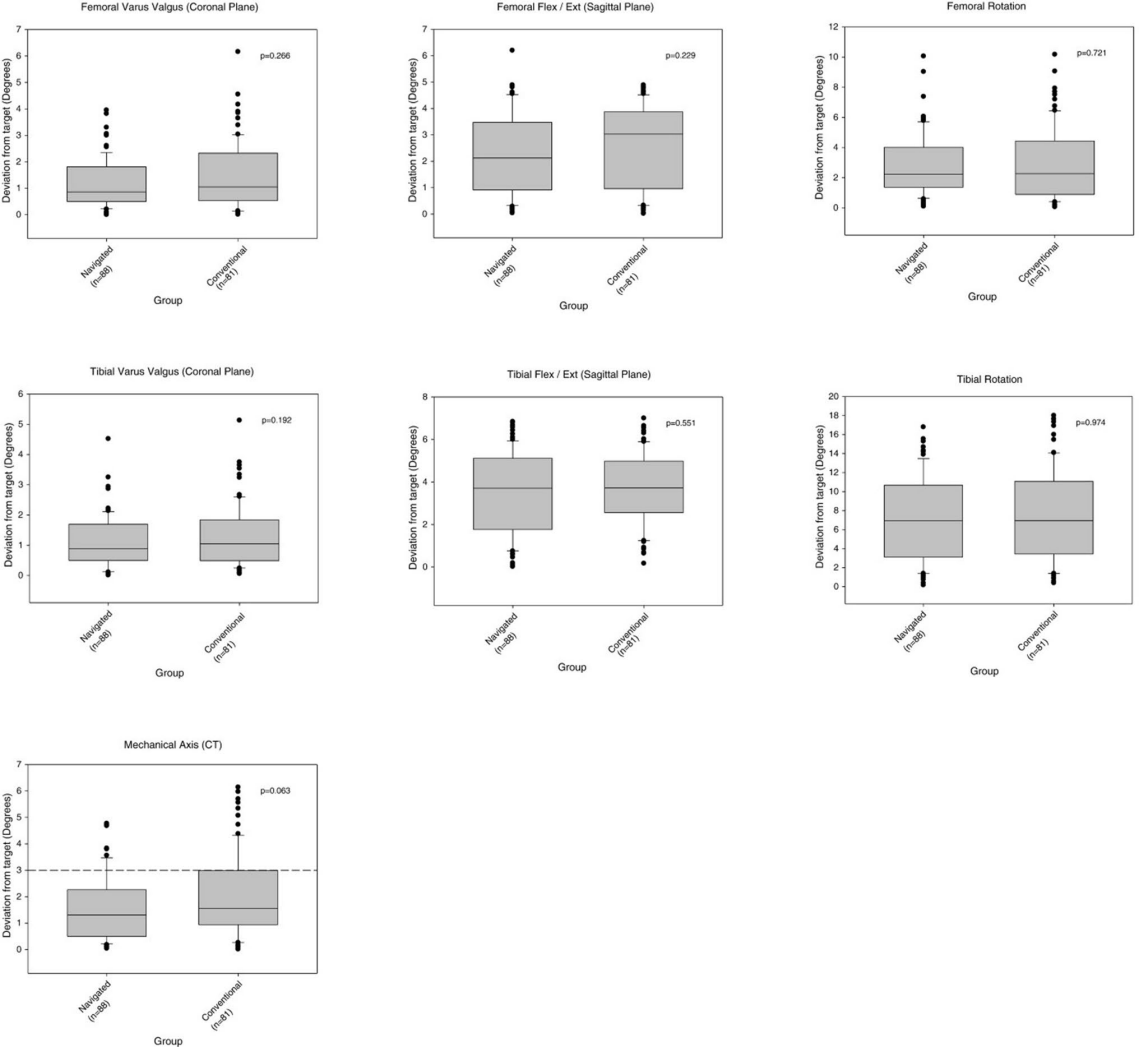
Comparison of post-operative accuracy of component placement in each plane and post-operative mechanical axis alignment as measured from CT scans. Dotted line represents the 3° target window for the mechanical axis

All patients received a cemented posterior stabilized NexGen LPS Flex TKA (Zimmer, Warsaw, Indiana, USA). Participants randomized to the conventional group received a TKA implanted using standard instrumentation, whereas those randomized to the navigated group had surgery using the iNav portable EM navigation system (Zimmer GmbH, Winterthur, Switzerland and Medtronic, Minneapolis, MN, USA). A standard process of joint registration maps the surface anatomy of the joint. All surgeries including joint surface registration were carried out by, or under the direct supervision of, one of two knee arthroplasty surgeons. Alignment targets were similar in both groups with a neutral HKAA and the aim to implant both femur and tibial components perpendicular to this in the coronal plane. There was no statistically significant difference in post-operative component alignment between the two groups (Fig. 2) [2]. HKAA for both groups was: navigated TKA = 179.8 ± 2.0° (175.2–184.7°) and conventional TKA = 179.7 ± 2.5° (173.8–185.9°), with 92% of navigated and 85% of conventional TKA patients achieving 180.0° ± 3.0° and 40% of navigated and 26% of conventional TKA patients achieving 180.0 ± 1.0° as measured by CT [2].

Ligament balancing was carried out using clinical assessment during the surgical procedure in both groups. In the navigated group, additional information was provided by the system with real-time feedback on the gap in mm between the femoral and tibial component and of the overall HKAA during varus and valgus stress.

### Patient-reported outcome measures and revisions

Participants who were still alive 10 years after surgery were followed up (navigated TKA; *n* = 41 and conventional TKA; *n* = 37, Fig. 1), with clinical assessments by a blinded independent assessor; range of motion (ROM) was determined using a hand-held goniometer, and knee-specific outcome measures included the American Knee Society Score (AKSS) and Oxford Knee Scores (OKS) and the SF-36 score used as a general health measure (both physical and mental). Revision surgery was also assessed by analysing the Scottish National Picture Archiving and Communication System. This image archiving system stores all imaging for patients undertaken in the NHS in Scotland since 2008. It acts as a valuable resource for identifying patients lost to follow-up who have undergone revision surgery in other NHS hospitals in Scotland which may have not been identified. This system does not store any imaging carried out in the private health care system, but private health insurance is held by only 8% of the Scottish population [26].

### Statistics

The primary outcome measure for this study was alignment within 3° of neutral mechanical alignment three months after surgery. To detect a difference of this magnitude with a power of 90% at alpha = 0.05, the initial power calculation indicated that we required 82 patients per group, 164 in total. The data presented in this manuscript represents an analysis of secondary outcome measures over the course of the study. Paired t-tests were performed to compare the change in each outcome measure from pre-operatively to 10 years, as well as comparing the difference between navigated and conventional groups overall at 10 years (Table 1). In addition to this, a Kaplan Meier survivorship graph has been created to compare the all-cause revision rates between navigated and conventional TKA surgeries which was analysed using a Mantel-Cox log-rank test on GraphPad Prism (version 6). To determine the differences between treatment allocations longitudinally, univariate general linear regression was used to model treatment allocation over time for each variable (IBM SPSS version 28).

**Table 1:** Patient-reported outcome measures pre-operatively and 10 years following navigated or conventional total knee arthroplasty

	Conventional (*n* = 98)		*p* value	Navigated (*n* = 101)		*p* value	10-year comparison *p* value
	Pre-op (*n* = 98)	10-year (*n* = 37)		Pre-op (*n* = 101)	10-year (*n* = 41)		
Gender (M/F)	38/60	15/22	0.846	46/55	21/20	0.581	0.372
Side (R/L)	50/48	22/15	0.441	50/51	16/25	0.272	0.111
Age at time of surgery, mean (SD)	64.9 (9.3)	64.5 (9.1)	0.822	65.3 (10.0)	65.1 (9.9)	0.913	0.317
OKS, median (Q1–Q3)	16.0 (11.0–20.3)	38.0 (31.0–41.0)	< 0.001	16.0 (11.0–20)	41.0 (29.0–44.0)	< 0.001	0.349
AKSS Knee, median (Q1–Q3)	37.0 (30.0–50.4)	90.0 (82.0–97.0)	< 0.001	38.0 (32.0–51.0)	95.0 (65.5–99.0)	< 0.001	0.358
AKSS Function, median (Q1–Q3)	47.5 (40.0–50.0)	75.0 (55.0–80.0)	< 0.001	50.0 (40.0–55.0)	80.0 (50.0–82.5)	< 0.001	0.906
SF36 Physical, median (Q1–Q3)	30.9 (25.5–35.0)	40.0 (23.1–66.9)	< 0.001	29.4 (22.5–38.8)	44.4 (27.8–71.4)	< 0.001	0.578
SF36 mental, median (Q1–Q3)	45.7 (31.3–56.4)	60.5 (48.5–81.3)	< 0.001	42.1 (33.0–56.3)	67.6 (43.3–82.6)	< 0.001	0.668
Flexion, mean (SD)	111.1 (14.6)	114.9 (12.3)	0.162	111.5 (10.7)	119.1 (9.1)	< 0.001	0.231
Extension, mean (SD)	5.7 (6.2)	0.4 (1.4)	< 0.001	5.8 (6.3)	0.3 (1.2)	< 0.001	0.837
Range of Motion, mean (SD)	105.3 (17.6)	114.5 (12.9)	0.004	105.6 (12.7)	118.8 (9.5)	< 0.001	0.253

Paired t tests comparing pre-op and ten year outcomes in each group, and two-tailed *t* tests were used comparing ten year outcomes between navigated and conventional groups were performed using GraphPad Prism v6.0. Data shown represents median (and range) or Mean (and standard deviation), and a *p* value of < 0.05 was deemed significant

## Results

### Clinical outcomes

Over the 10-year period, there were 23 deaths (22.8%) and 37 patients (36.6%) who withdrew or were lost to follow-up in the navigation group, and 30 deaths (30.6%) and 31 patients (31.6%) who withdrew or were lost to follow-up in the conventional group (Fig. 1).

The pre-operative demographics and pre-operative PROMs for participants available at 10-year clinical follow-up are shown in Table 1. We have previously reported that there was no statistically significant difference in participant demographics for all participants pre-operatively, and this remains the case for those patients who provided PROMs at 10 years. There was no statistically significant difference in any of the clinical outcomes between the navigated and conventional TKA cohorts at ten-year follow-up. However, clinically relevant and statistically significant improvements were made in all PROMs measured (with the exception of flexion but not overall range of motion; *p* = 0.162) from pre-operative to 10 years following surgery (Table 1).

**Fig. 3:**
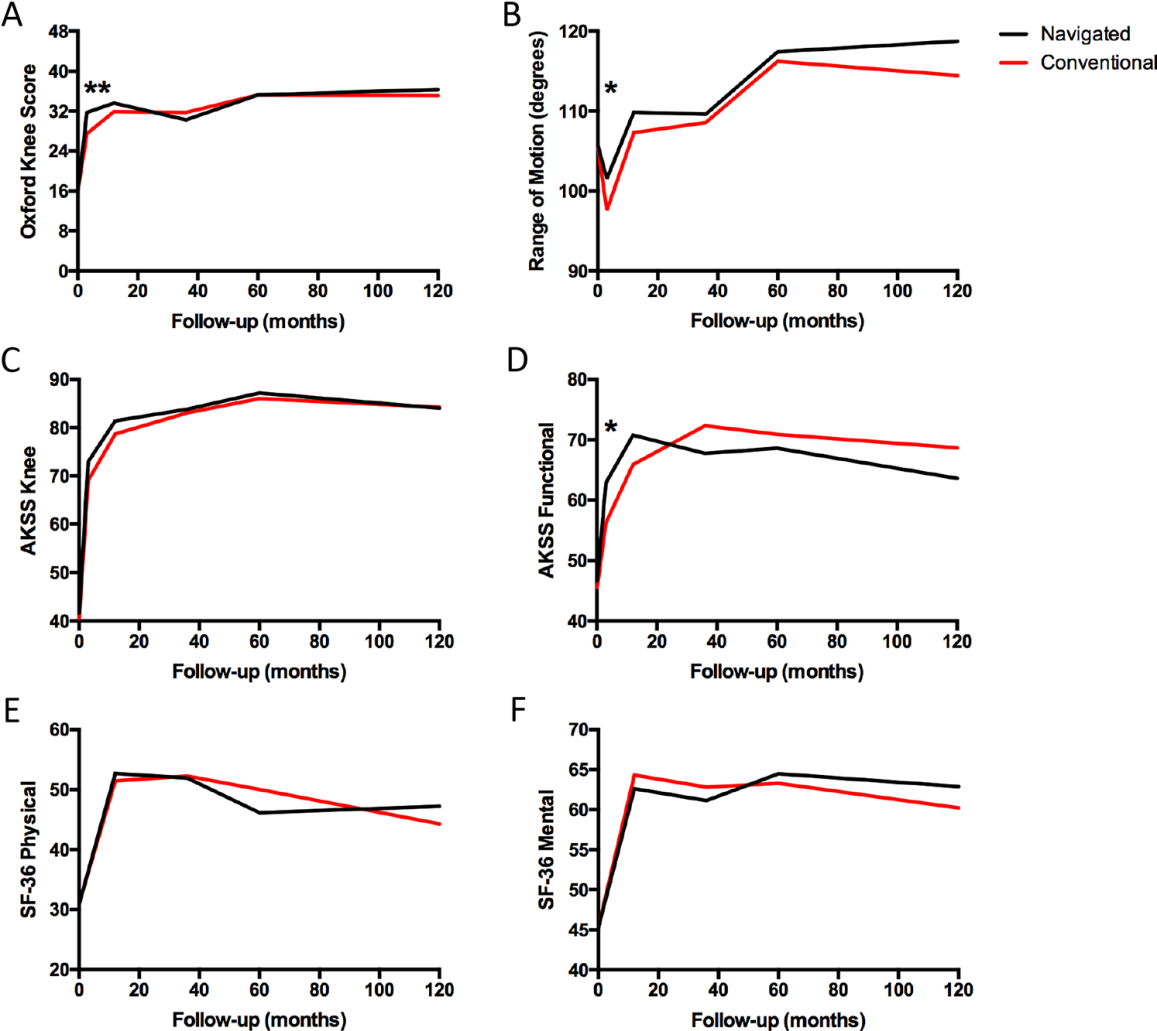
Mean Oxford Knee Score (**A**), Range of Motion (**B**), American Knee Society Scores for Knee Symptoms (**C**) and Function (**D**), and SF-36 Physical (**E**) and Mental (**F**) patient-reported outcomes over 10 years. *p < 0.05, and **p < 0.01

The PROMs for all patients remaining in the study from pre-operatively up to the completion of the study at 10 year follow-up provide evidence that navigated and conventional TKA provide similar long-term outcomes (Fig. 3 and Table 2). Interestingly, patients that received navigated TKAs had improved OKS, AKSS and ROM at 3 months following surgery compared to conventional TKA (*p* = 0.002, *p* = 0.032, and *p* = 0.05, respectively). However, the differences were only observed at 3 months post-op and from 1 to 10 years following surgery both interventions had equivalent outcomes.

**Table 2:** Patient reported outcome measures at all timepoints

		Timepoint					
		Pre-op	3 month	1 year	3 year	5 year	10 years
OKS, mean (SD)	Conventional	16.2 (6.4)	27.5 (10)	31.9 (10.6)	31.7 (11.3)	35.2 (9.5)	35.1 (9.7)
	Navigated	16 (6.3)	31.7 (8.8)	33.6 (8.9)	30.3 (11.3)	35.3 (9.3)	36.3 (9.7)
	*p* value	0.891	0.002**	0.217	0.412	0.990	0.553
AKSS, knee mean (SD)	Conventional	40.5 (16.3)	69.1 (17.6)	78.7 (16.9)	83.1 (18.3)	86.1 (17.5)	84.2 (17.4)
	Navigated	41.7 (13)	72.9 (15.1)	81.4 (12.7)	83.8 (14)	87.2 (17.1)	84.1 (18.8)
	*p* value	0.594	0.110	0.280	0.814	0.691	0.956
AKSS, function mean (SD)	Conventional	45.7 (14.5)	56.3 (20.2)	65.9 (22.9)	72.3 (20.9)	70.9 (22.7)	68.6 (22.3)
	Navigated	46.7 (15.4)	62.9 (18.1)	70.7 (20.9)	67.7 (21.2)	68.6 (21.2)	63.6 (31.2)
	*p* value	0.678	0.032*	0.123	0.275	0.538	0.28
ROM, mean (SD)	Conventional	105.4 (17.6)	97.6 (17.8)	107.3 (14.5)	108.6 (15.9)	116.2 (12.0)	114.5 (12.9)
	Navigated	105.7 (12.8)	101.5 (12.1)	109.8 (12.7)	109.6 (11.6)	117.4 (11.3)	118.8 (9.5)
	*p* value	0.881	0.050*	0.224	0.707	0.643	0.182
SF-36 physical, mean (SD)	Conventional	31.1 (10.3)	–	51.0 (25.9)	52.2 (29.4)	50.0 (24.4)	44.3 (26.4)
	Navigated	30.9 (12.5)	–	53.1 (26.4)	51.9 (27.1)	46.2 (26.8)	47.2 (26.6)
	*p* value	0.963	–	0.565	0.949	0.346	0.574
SF-36 mental, mean (SD)	Conventional	45.4 (16.7)	–	64.3 (23.3)	62.8 (23.9)	63.3 (25.5)	60.2 (22.5)
	Navigated	45.3 (17.5)	–	62.6 (24.8)	61.1 (22.3)	64.5 (24.6)	62.9 (21.6)
	*p* value	0.979	–	0.621	0.729	0.757	0.596

*p < 0.05, and **p < 0.01

### Survivorship

**Fig. 4:**
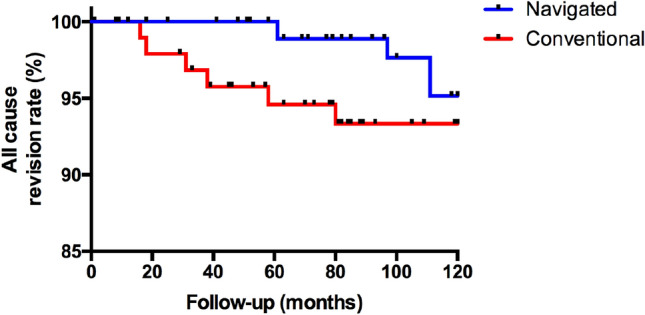
Kaplan–Meier survival curve with 95% Confidence Intervals for Total Knee Arthroplasty revision rate at 10 years following surgery comparing navigated (4.0%—4/101 patients) versus conventional surgery (6.1%—6/98 patients)

Table 3 demonstrates the re-interventions required for participants in each cohort at a 10-year follow-up. Revision was defined according to the National Joint Registry for England and Wales (NJR) as any case where a component of an arthroplasty is either removed, modified or added at a subsequent procedure [25]. There were six revisions in the conventional TKA and four revisions in the navigated TKA. In addition, one patient in the conventional TKA cohort required a manipulation under anaesthetic for stiffness at five-months post-operatively, with no revision required thereafter. Using this definition, there was no statistically significant difference in all cause revision between the two groups at 10 years follow-up (Fig. 4; 4.0% EM navigation vs 6.1% conventional group, *p* = 0.429). Furthermore, when analysing revisions excluding infection, there was no statistically significant difference between the two groups at 10 years follow-up (3.0% EM navigation vs 4.1% conventional group, *p* = 0.591).

**Table 3:** All cause re-interventions at ten-year follow-up

	Conventional (*n* = 98)	Navigated (*n* = 101)
Aseptic loosening	1	1
Early infection (< 3 months of index surgery)	1	0
Late infection (> 3 months from index surgery)	1	1
Instability	1	1
Patellofemoral resurfacing without implant exchange	2	1
Manipulation under anaesthesia for stiffness	1	0

## Discussion

This prospective study provides long-term follow-up results of a randomised controlled trial comparing navigated and conventional TKA. Previously, we have demonstrated at 5 years after surgery that there was a trend towards a higher revision rate in the conventional TKA cohort [4]. The ten-year results, however, show that there is no discernible difference in all-cause revision or PROMS between the groups at this time point. Furthermore, when we excluded revisions secondary to infection, and analysed causes of revision that might be influenced by CAS, we were still unable to identify any significant difference in implant survivorship between the two groups.

The potential for advanced health technologies to improve a range of outcome measures has led to their increasing use in surgery. It was estimated that in the United Kingdom (UK) and the United States of America (USA), 3–5% of TKA are by CAS [19]. By contrast, in Australia, the rate of CAS navigation has increased from 2.4% in 2003 to 32% in 2019 [1]. If the trend toward increased use of CAS navigation in Australia continues in the UK and USA, the health economics consequences may become an increasingly important factor. Due to competing demands from high-cost interventions, there is now a greater focus on delivering value in healthcare. There is a collective responsibility to meet patient expectations and ensure that additional costs incurred with the use of high-cost healthcare interventions are justified by their delivery of improved health outcomes. A recent scoping review identified several studies which suggested that CAS TKA may provide a cost-effective solution in TKA [27]. In one such study in a USA healthcare model, centres that perform a case volume of 250 TKAs per year would require a reduction of annual revision rates of 2% per annum over a 20 year period for CAS to be cost effective. This reduction in annual revision rate increased to 13% per annum over 20 years for centres which perform 25 TKAs per year [25]. Therefore, CAS is less likely to be a cost-effective investment in healthcare improvement in centres with a low volume of joint replacements, where its benefit is most likely to be realized [8, 25]. Whilst these studies suggest it may be cost-effective technology for centres with a higher volume of joint replacements, where the decrease in the rate of revision needed to make the investment cost-effective is modest, they assume that there is a reduction in revision rate associated with CAS TKA, something we have been unable to demonstrate in our study to date.

The largest study examining computer navigation in TKA arises from prospectively collected data in the Australian National Joint Registry. In this study, the authors compared the cumulative percent revision of 44,473 CAS TKA vs 270,545 conventional TKAs over a 9-year follow-up period. For the subgroup of patients < 65 years of age, there was a significant decrease in the revision rate in the CAS TKA group compared to the conventional TKA group (6.3 vs. 7.8%, *p* = 0.001) [5]. However, this study did not account for surgeon grade, volume or experience, patient variables or implant design which may have had an influence on the outcomes demonstrated. Several other studies have demonstrated conflicting evidence to this, showing no benefit, and a Norwegian registry study demonstrated worse survivorship outcomes with CAS TKA [7].

This study addresses the use of navigation in mechanically aligned TKA, and demonstrates no significant long-term advantage in the use of navigation utilising this philosophy. There is an increasing interest in alternative alignment strategies such as kinematic alignment, which aim to reproduce the patient’s pre-disease anatomy [23]. The theoretical advantage of this technique is a more naturally balanced knee replacement but it does result in a significant proportion of TKAs with tibial components implanted in severe varus (> 5°), which may influence long-term implant survivorship [11, 30]. RCTs comparing mechanical alignment and kinematic alignment suggest equivalent or improved PROMs with kinematic alignment but are limited by low participant numbers and limited follow-up to adequately demonstrate the risk of implant failure [22]. All RCTs comparing mechanical and kinematic alignment have relied either on computer navigation or patient-specific instrumentation to ensure precise implantation of particularly kinematically aligned TKAs, and the role of CAS in kinematically aligned TKAs to improve outcomes remains an area for further research.

There are limitations to our study. The study was performed in a high-volume arthroplasty centre with experienced knee surgeons, and this may explain the failure to demonstrate improved alignment seen in other studies with CAS TKA when compared to conventional TKA. Whilst this may skew the results in favour of conventional TKA, it is important to note that those studies demonstrating better alignment in CAS TKA have not been able to demonstrate improved survivorship or PROMS [13, 21]. Furthermore, the overall ten-year revision rate demonstrated in the NJR for this implant is 3.44% (95% confidence interval 3.33–3.56%), and represents a cumulative value of all surgeons inputting data irrespective of surgical volume [17]. This is comparable with the revision rate demonstrated within our conventional group, suggesting that this is representative of the overall practice. A further limitation of this study is the high rate of patients with incomplete PROMS data due to loss of follow-up (34.7% and 29.3% in the EM navigated and conventional groups, respectively). This is likely a consequence of the long-term nature of the study results and reflects attrition and mortality often seen in ten-year follow-up studies, with 30.6% and 22.8% of patients deceased in the conventional and navigated groups, respectively.

Crucially, however, we are confident that any revisions carried out in the NHS in Scotland would have been identified by our X-ray review on the National PACS. The numbers of patients in Scotland with private medical insurance is low (approximately 8.5%) and so it is highly unlikely that revisions were carried out in the private sector in any great numbers in Scotland [26]. In addition, our local hospital catchment area has high levels of social deprivation as measured by the Scottish Index of Multiple Deprivation, and although the percentage of our catchment area population with private medical insurance is unknown, we suspect that rates will be even lower than the Scottish average [24]. In addition, internal migration rates (moving between local authority areas) are less than 1% per annum in an age-matched population in the United Kingdom [20]. It is unlikely, therefore, that many, or indeed any, other patients in the study underwent revision in the private sector or outside Scotland within the study period.

When the study protocol was developed, the study was powered to detect a difference in the primary outcome of radiographic limb alignment between the two cohorts. Therefore, we accept that the study is underpowered to detect all but large differences in survivorship. To the best of our knowledge, all RCTs comparing EM navigation with conventional TKA are powered to detect either a differences in PROMS or precision of alignment and our study is comparable in size and findings to previously published RCTs [13, 21]. A post hoc power calculation performed, using a liberal assumption of a clinically significant 50% reduction in risk of revision at 10 years with the use of CAS powered at 80% would require approximately 1600 patients to detect a statistically significant difference. Whilst no RCTs examining CAS to date have been able to recruit this number of patients, this may be possible with the use of a meta-analysis, to which our study will help to contribute.

In conclusion, this study has shown no difference in revision rates or clinical outcomes comparing EM navigated versus conventional TKA at ten year follow-up. Whilst further follow-up data will be collected, the expected mortality rate makes it unlikely that a difference in revision rates will reach statistical significance in the future. In the setting of an experienced knee arthroplasty surgeon, it is difficult to justify the additional costs of CAS in TKA surgery.
